# Birthweight, gestational age and familial confounding in sex differences in infant mortality: a matched co-twin control study of Brazilian male-female twin pairs identified by population data linkage

**DOI:** 10.1093/ije/dyab242

**Published:** 2021-11-29

**Authors:** Lucas Calais-Ferreira, Marcos E Barreto, Everton Mendonça, Gillian S Dite, Martha Hickey, Paulo H Ferreira, Katrina J Scurrah, John L Hopper

**Affiliations:** Centre for Epidemiology and Biostatistics, Melbourne School of Population and Global Health, University of Melbourne, Melbourne, VIC, Australia; Centre for Adolescent Health, Murdoch Children’s Research Institute, Melbourne, VIC, Australia; AtyImoLab, Computer Science Department, Federal University of Bahia, Salvador, Brazil; Department of Statistics, London School of Economics and Political Science, London, UK; AtyImoLab, Computer Science Department, Federal University of Bahia, Salvador, Brazil; Centre for Epidemiology and Biostatistics, Melbourne School of Population and Global Health, University of Melbourne, Melbourne, VIC, Australia; Genetic Technologies Ltd., Melbourne, VIC, Australia; Royal Women’s Hospital, Melbourne, VIC, Australia; Department of Obstetrics and Gynaecology, University of Melbourne, Melbourne, VIC, Australia; Charles Perkins Centre Musculoskeletal Hub, Faculty of Medicine and Health, University of Sydney, Sydney, NSW, Australia; Centre for Epidemiology and Biostatistics, Melbourne School of Population and Global Health, University of Melbourne, Melbourne, VIC, Australia; Centre for Epidemiology and Biostatistics, Melbourne School of Population and Global Health, University of Melbourne, Melbourne, VIC, Australia

**Keywords:** Twins, infant, neonatal, mortality, sex differences, birthweight, familial confounding, gestational age, data linkage, administrative data

## Abstract

**Background:**

In infancy, males are at higher risk of dying than females. Birthweight and gestational age are potential confounders or mediators but are also familial and correlated, posing epidemiological challenges that can be addressed by studying male-female twin pairs.

**Methods:**

We studied 28 558 male-female twin pairs born in Brazil between 2012 and 2016, by linking their birth and death records. Using a co-twin control study matched for gestational age and familial factors, we applied logistic regression with random effects (to account for paired data) to study the association between male sex and infant death, adjusting for: birthweight, within- and between-pair effects of birthweight, birth order and gestational age, including interactions. The main outcome was infant mortality (0–365 days) stratified by neonatal (early and late) and postneonatal deaths.

**Results:**

Males were 100 g heavier and more at risk of infant death than their female co-twins before [odds ratio (OR)  = 1.28, 95% confidence interval (CI): 1.11–1.49, *P *=* *0.001] and after (OR = 1.60, 95% CI: 1.39–1.83, *P *<0.001) adjusting for birthweight and birth order. When adjusting for birthweight within-pair difference and mean separately, the OR attenuated to 1.40 (95% CI: 1.21–1.61, *P *<0.001), with evidence of familial confounding (likelihood ratio test, *P *<0.001). We found evidence of interaction (*P *=* *0.001) between male sex and gestational age for early neonatal death.

**Conclusions:**

After matching for gestational age and familial factors by design and controlling for birthweight and birth order, males remain at greater risk of infant death than their female co-twins. Birthweight’s role as a confounder can be partially explained by familial factors.

Key Messages

- Male twins are about 40% more likely to die in the first year of life than their female co-twins, despite being born 100g heavier, on average.
- The association between male sex and infant mortality remains after adjusting for familial confounders.
- Term births might increase the relative risk of neonatal death for males compared with female co-twins, although the absolute risk is decreased for both males and females.

## Background

Infant mortality, defined as deaths within the first year of life, remains a substantial public health problem globally. In 2019 alone, there were nearly four million infant deaths globally, and around 63% of those were neonatal deaths (within the first 28 days of life).^1^ Reports of the ‘male newborn disadvantage’ are not new in the literature.^2^ Males are at higher risk than females for neonatal morbidity and mortality despite being heavier at birth.^3^ This disadvantage is more evident for newborns with lower birthweight, as evidenced by a study in the USA which found that 22% [95% confidence interval (CI): 16–32] of boys and 15% (95% CI: 11–22) of girls who weighed less than 1500 g at birth died before hospital discharge.^4^

Studying sex differences in early life mortality is challenging, not least because birthweight and gestational age, which are likely to be potential confounders or mediators, are familial and correlated with each other.^5^ Therefore, it is still unclear whether and how birthweight and gestational age may partly explain the relationship between male sex and increased early life mortality. The extent to which these associations are confounded by familial factors, such as genetic variants and shared familial factors (including social determinants of health), is largely unknown. Although very rare in the literature, studies of male-female twin pairs present an opportunity to address this research gap.

A co-twin control study design^6^ based on male-female twin pairs can investigate a sex association while matching perfectly for age, year of birth and gestational age, as well as for pair-specific maternal characteristics, one-half of all autosomal genetic factors (on average), and environmental confounders common to the twins. Male-female pairs are the only type of twin pair discordant for sex and are as common as same-sex dizygotic pairs. Paired designs protect from bias due to uncontrolled confounding arising from factors that are common to family members and have a causal effect on both the exposure and the outcome.^7^

Random-effects models can be used to estimate the association between male sex and infant mortality while controlling for unmeasured familial factors and measured covariates that differ between twins in a pair. This is important because birthweight is influenced by maternal and genetic factors.^8^ A more general model allows for the estimation of between-pair as well as within-pair associations using random-effects logistic regression, including adjustment for measured and unmeasured (familial) risk factors shared between twins in a pair.^9–11^ This is a critical step for assessing and adjusting for familial confounders, which may also provide valuable information for causal inference.^12^

We have designed a matched opposite-sex co-twin control study to investigate the association between male sex and infant mortality while matching for gestational age, and unmeasured maternal and familial factors, and adjusting for birthweight and birth order as measured covariates. We applied deterministic data linkage to create a whole-of-population prospective cohort of male-female twin pairs by linking their birth and death records available from national health administrative databases in Brazil, and then studied this cohort to address our study objectives.

## Methods

The University of Melbourne's Human Research Ethics Committee (HREC) waived the need for ethics approval for this study, due to the use of unidentifiable secondary data freely available in the public domain.

### Data linkage and selection criteria

We used population-complete databases publicly available from the Brazilian population health databases system called Datasus.^13^ Initially, we used the birth records from the Brazilian live birth information system (Sistema de Informações sobre Nascidos Vivos—SINASC) database to extract a dataset of 15 125 061 babies born in all states of Brazil over 2012–16. We then selected 303 379 records with the type of pregnancy recorded as ‘double’ to indicate a twin pregnancy, including both same-sex and opposite-sex twins. Next, we linked the selected twins' birth records to Brazil's mortality information database (Sistema de Informações sobre Mortalidade—SIM) with all death records within 1 year of birth over 2012–17, so that all newborns would have been followed for up to 1 year.

This linkage process was entirely deterministic, performed by a straightforward linkage using the birth record number from twins in SINASC to all death records in SIM within 1 year of birth over 2012–17 with this unique identifier. We included all death records (not only twins) to avoid missing deaths that were not coded for a ‘double’ pregnancy due to potential coding errors. The birth record number was not present in 31% of death records. Given our interest in relative differences between males and females, we conducted a sensitivity analysis and found no evidence that proportions of males and females in death records of twins with the birth identifier were different from those records without the identifier (chi square test, *P *=* *0.093). Supplementary Table S1, available as Supplementary data at *IJE* online, presents proportions of outcomes for all twins (paired and unpaired) by year. Death data did not include stillbirths.

After linking birth and death records, we developed a bespoke algorithm to deterministically match twins to one another based on maternal, pregnancy- and birth-related variables that should have been reported to be the same for both members of twin pairs. The list of attributes used in the algorithm is presented in Supplementary Table S2, available as Supplementary data at *IJE* online. Our algorithm was able to match 208 044 (68.6%) of all the twin births into pairs, with increasing success from 64% (2012) to 71% (2016). Pairing results are shown in Supplementary Table S3, available as Supplementary data at *IJE* online. Due to the type of delivery being included in the algorithm as a matched variable to link twins into pairs, twins from pairs who were discordant for the type of delivery were excluded from the study. We also present results from sensitivity analyses for comparing paired and unpaired records to assess potential bias (Supplementary Tables S4 and S5, available as Supplementary data at *IJE* online).

**Figure 1:**
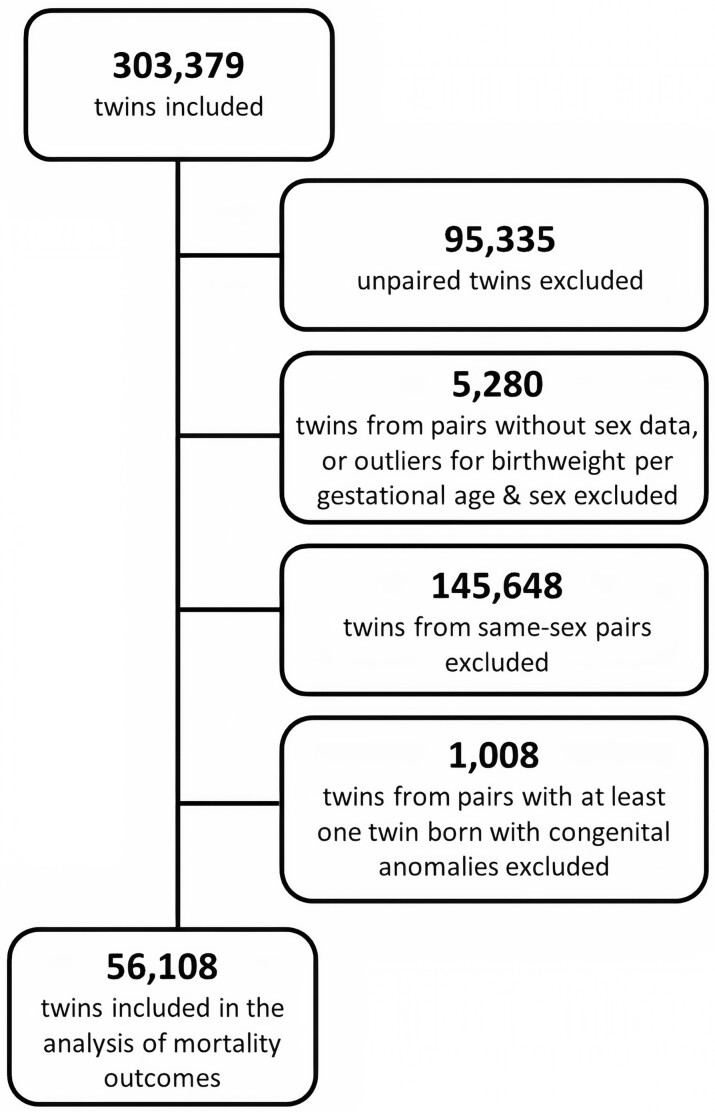
Flowchart of study’s selection criteria

We excluded 75 records with missing data for the sex variable and 4393 outliers with standardized scores of birthweight for gestational age and sex outside the range of −3.29 to 3.29, using Tukey's methodology,^14^ and removed 812 twins from incomplete pairs, resulting in a total of 202 764 twins in complete pairs. From those, we excluded 145 648 same-sex twins, resulting in 57 116 male-female twins in 28 558 pairs in the study. The analyses of mortality outcomes were restricted to newborns without congenital anomalies, due to the higher risk of early death for newborns with such malformations,^15^^,^^16^ leaving 56 108 twins in 28 054 complete pairs. A selection criteria flowchart is presented in Figure 1.

### Exposures

Shared characteristics within twin pairs included maternal age (years), parity at birth, gestational age (weeks), preterm (binary, <37 weeks of gestation as per WHO guidelines),^17^ number of prenatal consultations, type of delivery (caesarean or normal), marital status, race and education. Individual characteristics were sex (the main exposure), birthweight (continuous, per 100 g), low birthweight (binary, less than 2500 g, as per WHO guidelines)^18^ and birth order (binary). Within-pair differences and within-pair means of birthweight were also used.

### Outcomes

Birth outcomes were congenital anomalies detected at birth and Apgar scores at the first and fifth minutes (Apgar1 and Apgar5) scored from 1 to 10 and then categorised as low if <7. Following WHO guidelines,^19^ infant mortality was defined as death in the first year and was stratified into early neonatal deaths (death in the first 7 complete days), late neonatal deaths (death within 8 and 28 complete days) and postneonatal deaths (death in Days 29 to 365). There were some missing data: 1% for birth order, 1.2% for Apgar1, 1.2% for Apgar5 and 1.4% for congenital anomalies. Twins pairs with missing data for only one twin were included in the analyses.

### Statistical analysis

First, we calculated descriptive statistics for maternal characteristics of the twin pairs (Table 1) and tested for differences between males and females using paired t tests for the continuous variables: birthweight (per 100 g), Apgar1 and Apgar5 (Table 2). We calculated descriptive statistics for the mortality outcomes and McNemar’s tests for differences between males and females (Table 3).

**Table 1:** Maternal characteristics at birth

Variable	Mean or *n* (SD or %)
Mother’s age at birth, mean (SD)	29.6 (6.2)
Mother’s parity at birth, mean (SD)	1.4 (1.7)
Gestational age in weeks, mean (SD)	35.6 (3.0)
Gestational age (categorical), *n* (%)	
20–28 weeks	1556 (2.7%)
28–31 weeks	3224 (5.8%)
32–36 weeks	25 796 (45.2%)
37+ weeks	26 440 (46.3%)
Birthweight (100 g), mean (SD)	23.5 (5.6)
Low birthweight (<2500 g), *n* (%)	32 007 (56%)
Caesarean births, *n* (%)	49 228 (86.2%)
Race/skin colour	
Black	3784 (6.6%)
White	26 422 (46.3%)
Mixed	26 390 (46.2%)
Other	266 (0.5%)
Indigenous	188 (0.3%)
Undeclared	66 (0.1%)
Mother’s marital status, *n* (%)	
Single	18 870 (33.0%)
Married	25 668 (44.9%)
Widow	100 (0.2%)
Separated/divorced	840 (1.5%)
De facto	11 568 (20.3%)
Undeclared	70 (0.1%)
Mother’s education, *n* (%)	
0 years	308 (0.5%)
1–3 years	1684 (3.0%)
4–7 years	9330 (16.3%)
8–11 years	28 526 (49.9%)
12+ years^a^	17 064 (29.9%)
Undeclared	204 (0.4%)
Prenatal consultations, *n* (%)	
None	773 (1.4%)
1 to 3	2992 (5.3%)
4 to 6	13 016 (23.0%)
More than 7	39 915 (70.4%)

SD, standard deviation.

a Equivalent to high school completion.

**Table 2:** Mean within-pair differences in birthweight and Apgar scores, stratified by sex

	Males	Females	Difference (95% CI)	*P* ^a^
Birthweight (100g)	24	23	1.00 (0.95–1.04)	<0.001
Apgar1 score^b^	7.94	7.94	0 (−0.02–0.01)	0.7
Apgar5 score^b^	9.08	9.07	0.01 (−0.01–0.02)	0.5

a Paired t test; ^b^1–10 range.

**Table 3:** Mortality outcomes, stratified by sex

Outcome	Males	Females	*P* ^a^
Infant death, *n* (%)	783 (2.8%)	695 (2.5%)	0.001
Neonatal death, *n* (%)	635 (2.3%)	576 (2.1%)	0.014
Early neonatal death, *n* (%)	495 (1.8%)	433 (1.5%)	0.003
Late neonatal death, *n* (%)	140 (0.5%)	143 (0.5%)	0.853
Late infant death, *n* (%)	148 (0.5%)	119 (0.4%)	0.071

a McNemar test for paired difference between male and female co-twins.

Second, we investigated the association between male sex and the outcomes by estimating odds ratios (OR) for males (compared with females, the reference group) in: Model 1, an unadjusted random-effects model using maximum likelihood estimation; Model 2, which was the same as Model 1 but adjusted for birth order and birthweight; and Model 3, the same as Model 1 but adjusted for birth order and within- and between-pair differences of birthweight separately, by including both the within-pair absolute difference and the within-pair mean of birthweight in the models (Table 4).

**Table 4:** Association between male sex and birth and mortality outcomes, from unadjusted (1) and adjusted (2 and 3) models

Outcomes	Model 1^a^		Model 2^b^		Model 3^c^						LR test^d^ *P*
	OR (95% CI)	*P*	aOR (95% CI)	*P*	aOR (95% CI)	*P*	Birthweight Pair difference aOR (95% CI)	*P*	Birthweight Pair mean aOR (95% CI)	*P*	
Low Apgar1	1.02 (0.96–1.09)	0.541	1.22 (1.14–1.30)	<0.001	1.04 (0.97–1.11)	0.31	0.98 (0.96–1.00)	0.026	0.79 (0.78–0.80)	<0.001	<0.001
Low Apgar5	1.23 (1.06–1.43)	0.008	1.52 (1.31–1.76)	<0.001	1.22 (1.05–1.42)	0.011	0.96 (0.92–1.01)	0.115	0.70 (0.68–0.72)	<0.001	<0.001
Congenital anomalies	1.37 (1.14–1.65)	0.001	1.46 (1.21–1.76)	<0.001	1.60 (1.31–1.94)	<0.001	0.86 (0.82–0.90)	<0.001	0.95 (0.93–0.97)	<0.001	<0.001
Infant death	1.28 (1.11–1.49)	0.001	1.60 (1.39–1.83)	<0.001	1.40 (1.21–1.61)	<0.001	0.83 (0.79–0.87)	<0.001	0.68 (0.66–0.69)	<0.001	<0.001
Neonatal death	1.23 (1.04–1.45)	0.013	1.59 (1.36–1.87)	<0.001	1.35 (1.14–1.59)	<0.001	0.83 (0.78–0.88)	<0.001	0.64 (0.62–0.65)	<0.001	<0.001
Early neonatal death	1.35 (1.11–1.64)	0.002	1.72 (1.43–2.06)	<0.001	1.43 (1.18–1.73)	<0.001	0.84 (0.78–0.91)	<0.001	0.62 (0.60–0.64)	<0.001	<0.001
Late neonatal death	0.98 (0.77–1.24)	0.853	1.17 (0.91–1.50)	0.229	1.09 (0.84–1.41)	0.526	0.84 (0.77–0.92)	<0.001	0.76 (0.74–0.78)	<0.001	0.025
Postneonatal death	1.25 (0.98–1.60)	0.071	1.47 (1.15–1.89)	0.003	1.46 (1.13–1.89)	0.004	0.83 (0.77–0.90)	<0.001	0.82 (0.81–0.84)	<0.001	0.818

a Unadjusted model.

b Model adjusted for birth order and birthweight.

c Model adjusted for birth order, within-pair difference and within-pair mean of birthweight (per 100 g) separately.

d Likelihood ratio test of probability that Model 3 is a better fit for the data than Model 2, used in this context to test for presence of familial confounding. Mortality outcomes: infant death (within 0–365 days from birth), neonatal death (within 0–28 days from birth), early neonatal death (within 0–7 days from birth), late neonatal death (within 8–28 days from birth), postneonatal death (within 29–365 days from birth).

Twins were matched in each design. The difference between the models is the level of confounding adjustment, going from the more generic crude Model 1 to the more specific Model 3 which accounted for the familial aggregation in birthweight as a confounder. We used a likelihood ratio test (LR Test) to compare Models 2 and 3 to assess evidence of familial confounding.

To study interactions, we then fitted Model 4, which modelled the association between male sex and outcomes adjusting for birth order, birthweight pair difference, birthweight pair mean, gestational age and an interactive term between male sex and low birthweight, with ORs presented separately for twins with low (<2500 g) and normal (≥2500) birthweight. Model 5 adjusted for the same variables, but the interactive term was for male sex by preterm births, with separate results for preterm (<37 weeks) and term (≥37 weeks) births.

We studied interactions of male sex with birthweight pair mean and male sex with gestational age as continuous variables while adjusting for covariates for all mortality outcomes (Supplementary Tables S6 and S7, available as Supplementary data at *IJE* online, respectively). The Supplementary material also includes results of models assessing a potential interaction between birthweight pair mean with gestational age (Supplementary Table S8, available as Supplementary data at *IJE* online) and male sex with birth order (Supplementary Table S9, available as Supplementary data at *IJE* online), while adjusting for the same set of covariates.

Finally, we fitted additional models to investigate interactions of male sex with standardized birthweight per gestational age derived from twin-specific birthweight percentile charts in the literature,^20^ and for appropriate birthweight per gestational age defined as standardized birthweight per gestational age equal or above 0 (Supplementary Table S10, available as Supplementary data at *IJE* online). This was done as sensitivity analysis to understand whether the correlation between gestational age and birthweight would affect the sex interactions.

All analyses were conducted using Stata/SE 16.0, using the *xtlogit* command for fitting the random-effects models. The charts were created by using the *marginsplot* command. This study was reported according to the REporting of studies Conducted using Observational Routinely-collected health Data (RECORD) Statement.^13^

## Results

### Descriptive statistics

The mean age of the twins' mothers was approximately 30 years (Table 1). The mean gestational age was 36 weeks, 1 week below the clinically accepted threshold for term births.^17^ About half of all twins were preterm; 70% had seven or more prenatal consultations. Males were, on average, 100g heavier than their female co-twins at birth (*P *<0.001). There was no evidence of sex differences in Apgar1 and Apgar5 scores (Table 2). There were 1478 deaths observed in a total of 28 054 pairs included in the mortality analysis, being 783 (2.8%) deaths for males and 695 (2.5%) for females (Table 3).

### Birth and mortality outcomes

#### Unadjusted (1) and adjusted (2) models

There was a consistent increase in ORs from those estimated in the unadjusted Model 1 to Model 2 adjusted for birth order and birthweight in a single variable for all outcomes, suggesting that birthweight is a negative confounder of the sex associations (Table 4). After adjusting for birthweight and birth order, males were 60% more likely to die in the first year of life (infant death) compared with their female co-twins [adjusted odds ratio [aOR] = 1.60, 95% CI: 1.39–1.83, *P *<0.001), increasing from 1.28 (95% CI: 1.11–1.49, *P *=* *0.001) in the unadjusted model. The aOR for the association between male sex and neonatal mortality (0–28 days) was 1.59 (95% CI: 1.36–1.87, *P *<0.001) from the adjusted Model 2. This positive association was mainly driven by early neonatal deaths. For those, the adjusted OR was 1.72 (95% CI: 1.43–2.06, *P *<0.001). We found evidence of a sex association for postneonatal death (aOR = 1.47, 95% CI: 1.15–1.89, *P *=* *0.003) but not for late neonatal death (aOR = 1.17, 95% CI: 0.91–1.50, *P *=* *0.229) from the adjusted Model 2.

#### Within- and between-pair Model 3

Table 4 also shows the association between male sex and the stratified infant mortality categories after adjusting for the within-pair differences and means of birthweight. Using a likelihood ratio test (LR Test), we found evidence of familial confounding (LR Test, *P *<0.001), favouring Model 3 for all mortality outcomes except postneonatal death. However, the confidence intervals of estimates of the sex association overlapped between these models, with better precision in Model 3.

The aOR for early neonatal deaths attenuated from 1.72 (95% CI: 1.43–2.06, *P *<0.001) to 1.43 (95% CI: 1.18–1.73, *P *<0.001) between Models 2 and 3, suggesting that the effect of birthweight as a negative confounder can be partially explained by familial confounding. For infant death, an increase of 100 g in the birthweight pair mean was a stronger protective factor (aOR = 0.68, 95% CI: 0.66–0.69, *P *<0.001) than being 100g heavier than their co-twin (aOR = 0.83, 95% CI: 0.79–0.87, *P *<0.001).

#### Interactions

We found some evidence of an interaction between male sex and preterm status for neonatal death (*P *=* *0.014) and early neonatal death (*P *=* *0.033) (Model 5, Table 5). The odds of early neonatal death for males were nearly four times higher than for their female co-twins for term infants, although 95% confidence intervals were wide (aOR = 3.68, 95% CI: 1.54–8.82, *P *=* *0.003). In Model 4, we found evidence of interaction between male sex and low birthweight for low Apgar1 (*P *=* *0.003) and postneonatal death (*P *=* *0.008).

**Table 5:** Association between male sex and birth and mortality outcomes, with interactions of male sex with low birthweight (Model 4), and male sex with preterm status (Model 5)

Outcomes	Model 4^**a**^					Model 5^a^				
	Low birthweight (<2500 g)		Normal birthweight (≥2500 g)		Interactive term^**b**^	Preterm (<37 weeks)		Term (≥37 weeks)		Interactive term^c^
	aOR (95% CI)	*P*	aOR (95% CI)	*P*	*P*	aOR (95% CI)	*P*	aOR (95% CI)	*P*	*P*
Low Apgar1	1.11 (1.03–1.21)	0.01	0.88 (0.78–1.00)	0.055	0.003	1.07 (0.99–1.17)	0.079	0.96 (0.85–1.08)	0.474	0.114
Low Apgar5	1.32 (1.11–1.56)	0.002	0.93 (0.64–1.35)	0.696	0.096	1.33 (1.12–1.57)	0.001	0.86 (0.60–1.25)	0.434	0.036
Congenital anomalies	1.53 (1.20–1.94)	<0.001	1.73 (1.24–2.42)	0.001	0.554	1.77 (1.38–2.27)	<0.001	1.36 (1.01–1.83)	0.042	0.175
Infant death	1.50 (1.29–1.75)	<0.001	0.97 (0.57–1.63)	0.895	0.112	1.42 (1.21–1.65)	<0.001	1.62 (1.07–2.45)	0.023	0.553
Neonatal death	1.38 (1.16–1.64)	<0.001	1.84 (0.79–4.28)	0.157	0.513	1.31 (1.10–1.56)	0.003	3.07 (1.58–5.99)	0.001	0.014
Early neonatal death	1.48 (1.21–1.80)	<0.001	1.61 (0.54–4.77)	0.389	0.875	1.40 (1.14–1.71)	0.001	3.68 (1.54–8.82)	0.003	0.033
Late neonatal death	1.05 (0.81–1.37)	0.7	2.20 (0.58–8.32)	0.247	0.287	1.03 (0.79–1.35)	0.799	2.13 (0.78–5.81)	0.14	0.171
Postneonatal death	1.66 (1.26–2.20)	<0.001	0.60 (0.29–1.22)	0.159	0.008	1.62 (1.21–2.16)	0.001	0.98 (0.57–1.70)	0.945	0.11

a Adjusted for birth order, birthweight pair difference, birthweight pair mean, gestational age and interactive term.

b Male sex by low birthweight status.

c Male sex by preterm status.

**Figure 2:**
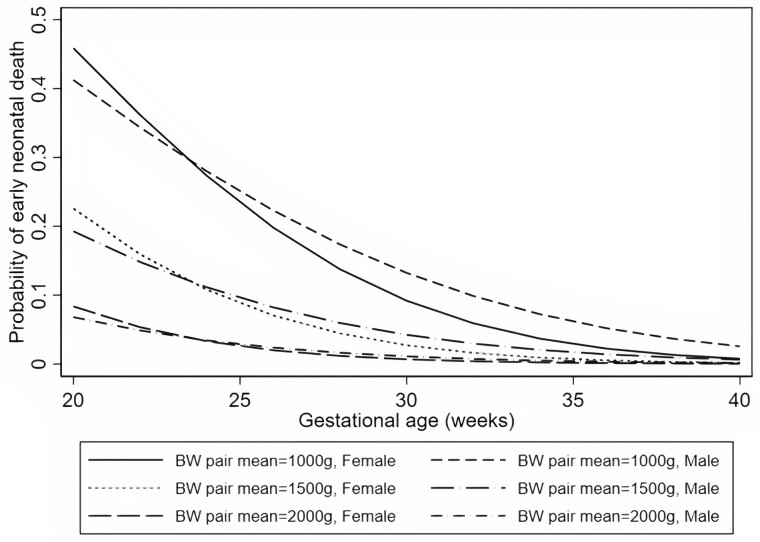
Probability of early neonatal death for males and females under three different scenarios for birthweight (BW) pair mean, with birth order fixed at ‘firstborn’ and birthweight pair difference fixed at 0. Model adjusted for birth order, gestational age, birthweight pair difference, birthweight pair mean, and an interactive term for sex and gestational age as a continuous variable

For early neonatal deaths, we found strong evidence of interaction between male sex and gestational age (*P *=* *0.001), and male sex and birthweight pair mean (*P *=* *0.001), both as continuous variables (Supplementary Tables S7 and S6, respectively). We plotted the probabilities of early neonatal death by gestational age for males and females under three different scenarios of birthweight pair mean (1000 g, 1500 g and 2000 g), while fixing birth order at ‘first born’ and birthweight pair difference at 0, and allowing for an interaction between male sex and gestational age (Figure 2).

Finally, we found evidence of interaction between gestational age and birthweight (as continuous variables) for all outcomes but postneonatal death (Supplementary Table S8). We did not find strong evidence of interaction between male sex and birth order (Supplementary Table S9) nor between male sex and the standardiezd birthweight variables per twin-specific charts (Supplementary Table S10).

## Discussion

We investigated the roles of birthweight, gestational age and familial confounding in the association between male sex and infant mortality by applying a twin design to study male-female twin pairs. Our results show that males were at substantially higher risk than females for low Apgar5, congenital anomalies and infant, neonatal, early neonatal and postneonatal deaths after adjusting for birth order and within- and between-pair estimates of birthweight separately. This was despite males being heavier at birth by 100g on average.

The overall proportion of infant mortality for boys compared with girls was greater than previously reported for the non-twin population,^3^^,^^21^ including a previous study conducted in Brazil.^22^ However, the 40% (95% CI: 1.21–1.61) additional risk of infant mortality for boys compared with their female co-twins found in our study, even after controlling for birthweight and familial confounding, was similar to that of other studies of opposite-sex newborn twin pairs from the USA and Israel.^23^^,^^24^ This suggests that the twin cohort generated from our method to ascertain twin pairs from their de-identified birth and death records appears to be largely representative of the twin population.

For the first time, we found that the within- and between-pair differences in birthweight were independent predictors of infant mortality when fitted separately (Model 4). Nonetheless, the attenuation from the OR of 1.60 (95% CI: 1.39–1.83) to 1.40 (95% CI: 1.21–1.61) provides an estimate of the magnitude of familial (negative) confounding related to birthweight in the association between sex and infant mortality. Our evidence that familial confounding is more important for early neonatal than later deaths has not been previously reported in the literature. Our study found that male sex predicts infant death independently of birth order and within- and between-pair differences in birthweight (which are also independent from each other).

We found strong evidence of an interaction between male sex and gestational age for early neonatal deaths. This is important, given the previously reported longer gestational age for opposite-sex twin pairs compared with same-sex twin pairs^25^ and suggestions of a ‘masculinizing effect’^26^ which would theoretically support the hypothesis of a disadvantage for females rather than an advantage for males. Matching opposite-sex twin pairs for gestational age and for unmeasured shared maternal factors, which can differ between male and female singletons, including reported factors such as maternal immunological responses and sex steroid concentrations,^3^ allowed us to estimate the sex association unconfounded by these factors.

Our study had some limitations related to contingencies in the data linkage process. The sensitivity analysis (Supplementary material) revealed differences between the twins matched and those not matched in pairs by our algorithm. We compared these groups as a linkage quality measure as suggested by others,^27^ and found that, in the excluded group (unpaired twins), infant mortality was higher overall but males had a very similar risk ratio to that of paired twins. Our approach attempted to have the highest possible level of sensitivity (true positives) for twin pairs, although this meant the exclusion of 31% (95 335/303 379) of the twin sample which did not have a match for all the attributes used in the matching algorithm. This strategy was used because our study design focused on paired analyses, where inference is made about the co-twin.

Given that around 30% of the mortality records did not have a unique identifier (birth number) available for linkage with birth records, we might have underestimated absolute mortality numbers. Differences in the quality of mortality records in Brazil depending on death location have been reported.^28^ Potential issues with the use of deterministic data linkage techniques to link birth to death records, compared with using probabilistic techniques, have also been previously discussed.^29^ Unfortunately, the latter was not possible in our data linkage process due to the use of de-identified data and the unavailability of additional linkable attributes in the death records database. The lack of available data on additional covariates, both shared and non-shared between the twin pairs, was also a limitation.

Our findings have clear implications for the Brazilian public health care setting, which is primarily guided by low birthweight and prematurity guidelines to determine eligibility for primary care interventions. For example, the ‘kangaroo method’,^30^ a public health intervention aiming to increase the physical contact of mothers with their low-birthweight newborn babies, might benefit from twin-specific guidelines that also considers sex differences in mortality. Increased birthweight for males compared with their female co-twins, on average, did not confer any protection from infant mortality. Our interaction analyses indicated that longer gestational age increases the relative risk of early neonatal deaths for males compared with their female co-twins, although the absolute risk is decreased for both males and females. Nonetheless, this finding should be considered in light of established benefits of longer twin gestations.^31^^,^^32^

Our study showed that boys are at particularly higher risk of death in the first 7 days of life compared with their female co-twins. We showed that the familial aggregation of birthweight should be properly considered when adjusting for this variable, by demonstrating the application of the ‘within-between’ method for the first time in twin pairs of the opposite sex. Our findings also advance the understanding of the interplay between birthweight, gestational age and familial confounding in the association between sex and infant mortality.

## Supplementary Data

Supplementary data are available at *IJE* online.

## Funding

L.C.F., P.H.F. and K.J.S. were funded by the National Health and Medical Research Council’s (NHMRC) Australian Centre of Excellence in Twin Research (APP1079102). M.E.B. is a Newton International Fellow Alumnus (Royal Society, UK) and holds grants from Google.org, NVIDIA, Bill & Melinda Gates Foundation, MRC (UK) and Wellcome Trust (UK). G.S.D. is employed by Genetic Technologies Ltd. M.H. was funded by an NHMRC Investigator Grant (APP1193838). J.L.H. is an NHMRC Senior Principal Research Fellow (APP1137349). None of the funding sources had any role in the preparation of this manuscript.

## Author contributions

L.C.F. co-designed the study and the analytical strategy, conducted the statistical analysis and drafted the manuscript. M.E.B. and E.M. co-designed the data linkage strategy, acquired the data and revised the manuscript. G.S.D., M.H. and P.H.F. co-designed the study, helped interpret findings and revised the manuscript. K.J.S. and J.L.H. co-designed the study and the analytical strategy, guided the statistical analysis, helped interpret findings and revised the manuscript. All authors approved the final version of this manuscript.

## Conflict of Interest

None declared.

## Supplementary Material

dyab242_Supplementary_Data
