# Supplementary material for: Birthweight, gestational age and familial confounding in sex differences in infant mortality: a matched co-twin control study of Brazilian male-female twin pairs identified by population data linkage
Source: Int J Epidemiol. 2021 Nov 29;51(5):1502–10. doi: 10.1093/ije/dyab242 (PMC9557851; doi:10.1093/ije/dyab242)
Supplement: dyab242_Supplementary_Data [file dyab242_supplementary_data.docx]

**SUPPLEMENTARY MATERIAL**

| **Table S1: Outcomes of all twin pairs by year** | | | | | | |
| --- | --- | --- | --- | --- | --- | --- |
|  | **2012** | **2013** | **2014** | **2015** | **2016** | **Total** |
| Total twins born (n) | 60 589 | 61 141 | 62 750 | 61 358 | 57 541 | 303 379 |
| Low birthweight (<2500g) | 35 669 (58.9%) | 36 230 (59.3%) | 37 125 (59.2%) | 36 254 (59.1%) | 34 223 (59.5%) | 179 501 (59.2%) |
| Males | 165 77 (55.0%) | 16 908 (55.9%) | 17 384 (55.9%) | 17 012 (55.7%) | 16 156 (56.1%) | 84 037 (55.7%) |
| Females | 19 092 (62.7%) | 19 322 (62.6%) | 19 741 (62.5%) | 19 242 (62.5%) | 18 067 (62.9%) | 95 464 (62.6%) |
| Low APGAR score at 1st minute (<7) | 8472 (14.0%) | 8313 (12.6%) | 8521 (13.6%) | 8072 (13.2%) | 7699 (13.4%) | 41077 (13.6%) |
| Males | 4380 (14.5%) | 4244 (14.0%) | 4414 (14.2%) | 4194 (13.7%) | 4004 (13.9%) | 21 236 (14.1%) |
| Females | 4092 (13.4%) | 4069 (13.2%) | 4107 (13.0%) | 3878 (12.6%) | 3695 (12.9%) | 19 841 (13.0%) |
| Low APGAR score at 5th minute (<7) | 2128 (3.5%) | 2152 (3.5%) | 2173 (3.5%) | 1985 (3.2%) | 1770 (3.10%) | 10 208 (3.4%) |
| Males | 1083 (3.6%) | 1169 (3.9%) | 1160 (3.7%) | 1057 (3.5%) | 932 (3.2%) | 5401 (3.6%) |
| Females | 1045 (3.4%) | 983 (3.2%) | 1013 (3.2%) | 928 (3.0%) | 838 (2.9%) | 4807 (3.2%) |
| Congenital anomalies | 702 (1.2%) | 640 (1.1%) | 704 (1.2%) | 708 (1.2%) | 688 (1.2%) | 3442 (1.2%) |
| Males | 388 (1.3%) | 336 (1.1%) | 397 (1.3%) | 367 (1.2%) | 370 (1.3%) | 1858 (1.3%) |
| Females | 314 (1.1%) | 304 (1.0%) | 307 (1.0%) | 341 (1.1%) | 318 (1.1%) | 1584 (1.1%) |
| Infant mortality (0-365 days) | 2260 (3.7%) | 2357 (3.9%) | 2415 (3.9%) | 2376 (3.9%) | 2236 (3.9%) | 11 644 (3.8%) |
| Males | 1178 (3.9%) | 1285 (4.3%) | 1291 (4.2%) | 1295 (4.2%) | 1201 (4.2%) | 6250 (4.1%) |
| Females | 1082 (3.6%) | 1072 (3.5%) | 1124 (3.6%) | 1081 (3.5%) | 1035 (3.6%) | 5394 (3.5%) |
| Neonatal mortality (0-28 days) | 1918 (3.2%) | 2001 (3.3%) | 2081 (3.3%) | 1979 (3.2%) | 1890 (3.3%) | 9869 (3.3%) |
| Males | 997 (3.3%) | 1096 (3.6%) | 1115 (3.6%) | 1081 (3.5%) | 1018 (3.6%) | 5307 (3.5%) |
| Females | 921 (3.0%) | 905 (2.9%) | 966 (3.1%) | 898 (2.9%) | 872 (3.0%) | 4562 (3.0%) |
| Early neonatal mortality (0-7 days) | 1518 (2.5%) | 1615 (2.6%) | 1659 (2.7%) | 1577 (2.6%) | 1474 (2.6%) | 7843 (3.6%) |
| Males | 793 (2.6%) | 915 (3.0%) | 914 (2.9%) | 870 (2.9%) | 797 (2.8%) | 4289 (2.8%) |
| Females | 725 (2.4%) | 700 (2.3%) | 745 (2.4%) | 707 (2.3%) | 677 (2.4%) | 3554 (2.3%) |
| Late neonatal mortality (8-28 days) | 400 (0.7%) | 386 (0.6%) | 422 (0.7%) | 402 (0.7%) | 416 (0.7%) | 2026 (0.7%) |
| Males | 204 (0.7%) | 181 (0.6%) | 201 (0.7%) | 211 (0.7%) | 221 (0.8%) | 1018 (0.7%) |
| Females | 196 (0.6%) | 205 (0.7%) | 221 (0.7%) | 191 (0.6%) | 195 (0.7%) | 1008 (0.7%) |
| Post neonatal mortality (29-365 days) | 342 (0.6%) | 356 (0.6%) | 334 (0.5%) | 397 (0.7%) | 346 (0.6%) | 1775 (0.6%) |
| Males | 181 (0.6%) | 189 (0.6%) | 176 (0.6%) | 214 (0.7%) | 183 (0.6%) | 943 (0.6%) |
| Females | 161 (0.5%) | 167 (0.5%) | 158 (0.5%) | 183 (0.60%) | 163 (0.6%) | 832 (0.6%) |

| **Table S2:** Variables used in deterministic linkage algorithm to match twins in pairs | |
| --- | --- |
| **Variable** | **Description** |
| LOCNASC | Location of birth (home, hospital or other) |
| CODESTAB | Code of hospital |
| CODISNT | Code of register installation |
| CODMUNNASC | Code of municipality |
| IDADEMAE | Mother’s age in years |
| ESTCIVMAE | Mother’s marital status |
| ESCMAE | Mother’s education in years |
| CODMUNRES | Code of municipality of mother’s address |
| GRAVIDEZ | Type of pregnancy (single, twin or higher order multiple) |
| PARTO | Type of birth delivery |
| DTNASC | Date of birth |
| CODMUNNATU | Code of municipality of mother’s birth location |
| DTNASCMAE | Mother’s date of birth |
| RACACORMAE | Mother’s self-reported race |
| CODUFNATU | Code of state of mother’s birth location |

| **Table S3: Pairing percentage by year** | | | | | | |
| --- | --- | --- | --- | --- | --- | --- |
|  | **2012** | **2013** | **2014** | **2015** | **2016** | **Total** |
| Total twins born | 60 589 | 61 141 | 62 750 | 61 358 | 57 541 | 303 379 |
| Pairing percentage | 63.6% | 67.1% | 70.2% | 70.8% | 71.3% | 68.6% |
| Paired male-male twins ^a^ | 13 684 | 14 658 | 15 654 | 15'598 | 14 660 | 45 912 |
| Paired female-female twins ^a^ | 14 004 | 14 886 | 15 804 | 15 878 | 14 658 | 46 340 |
| Paired male-female twins | 10 860 | 11 476 | 12 572 | 11 958 | 11 694 | 58 560 |
| **Total paired twins** | 38 548 | 41 020 | 44 030 | 43 434 | 41 012 | 208 044 |
| ^a^ Includes both monozygotic (MZ) and dizygotic (DZ) twins, while male-female pairs can be only DZ. | | | | | |  |

| **Table S4:** Proportions of birth and mortality outcomes of 303,379 twins, by pairing status | | | | | |
| --- | --- | --- | --- | --- | --- |
| **Outcome** | **Paired** |  | **Unpaired** |  | ***P*** *^a^* |
| Low APGAR1 | 12.8% |  | 15.2% |  | <0.001 |
| Low APGAR5 | 2.9% |  | 4.5% |  | <0.001 |
| Congenital anomalies | 1.2% |  | 1.3% |  | 0.001 |
| Infant death | 3.4% |  | 4.9% |  | <0.001 |
| Neonatal death | 2.9% |  | 4.2% |  | <0.001 |
| Early neonatal death | 2.2% |  | 3.4% |  | <0.001 |
| Late neonatal death | 0.6% |  | 0.8% |  | <0.001 |
| Post-neonatal death | 0.5% |  | 0.7% |  | <0.001 |
| ^a^ Chi-squared test |  |  |  |  |  |

| **Table S5:** Proportions of birth and mortality outcomes of 95,335 unpaired twins, by sex | | | | | |
| --- | --- | --- | --- | --- | --- |
| **Outcome** | **Males** |  | **Females** |  | ***P*** *^a^* |
| Low APGAR1 | 15.9% |  | 14.5% |  | <0.001 |
| Low APGAR5 | 4.8% |  | 4.1% |  | <0.001 |
| Congenital anomalies | 1.4% |  | 1.2% |  | 0.004 |
| Infant death | 5.3% |  | 4.4% |  | <0.001 |
| Neonatal death | 4.6% |  | 3.7% |  | <0.001 |
| Early neonatal death | 3.8% |  | 3.0% |  | <0.001 |
| Late neonatal death | 0.8% |  | 0.8% |  | 0.745 |
| Late infant death | 0.7% |  | 0.7% |  | 0.382 |
| ^a^ Chi-squared test |  |  |  |  |  |

| **Table S6:** Interactive terms between male sex and birthweight pair mean in the association between male sex and mortality outcomes, separately for strata of low and normal birthweight | | | | | | | | | | |
| --- | --- | --- | --- | --- | --- | --- | --- | --- | --- | --- |
| **Outcomes** |  | **Low birthweight  (<2500g)** | | **Normal birthweight (≥2500g)** | |  | | **All** | | |
|  |  | **Interactive term** ^a^  **(95% CI)** | ***P*** | **Interactive term** ^a^  **(95% CI)** | ***P*** | |  | | **Interactive term** ^a^  **(95% CI)** | ***P*** |
| Infant death |  | 1.03 (1.00–1.06) | 0.037 | 0.96 (0.77–1.19) | 0.690 | |  | | 1.02 (0.99–1.04) | 0.155 |
| Neonatal death |  | 1.02 (0.99–1.06) | 0.166 | 0.92 (0.65–1.30) | 0.625 | |  | | 1.03 (1.00–1.06) | 0.057 |
| Early neonatal death |  | 1.07 (1.03–1.11) | 0.001 | 1.12 (0.72–1.72) | 0.618 | |  | | 1.06 (1.02–1.10) | 0.001 |
| Late neonatal death |  | 0.98 (0.94–1.02) | 0.384 | 0.64 (0.36–1.14) | 0.131 | |  | | 0.99 (0.95–1.03) | 0.692 |
| Post neonatal death |  | 1.03 (0.99–1.08) | 0.152 | 1.07 (0.80–1.44) | 0.649 | |  | | 1.00 (0.96–1.03) | 0.838 |
| ^a^ adjusted for birth order, birthweight pair difference, birthweight pair mean, gestational age and male sex by birthweight pair mean (interactive term). | | | | | | | | | | |

| **Table S7:** Interactive terms between male sex and gestational age in the association between male sex and mortality outcomes, separately for strata of preterm and term births | | | | | | | | |
| --- | --- | --- | --- | --- | --- | --- | --- | --- |
| **Outcomes** |  | **Preterm births  (<37 weeks)** | | **Term births  (≥37 weeks)** | |  | **All** | |
|  |  | **Interactive term** ^a^  **(95% CI)** | ***P*** | **Interactive term** ^a^  **(95% CI)** | ***P*** |  | **Interactive term** ^a^  **(95% CI)** | ***P*** |
| Infant death |  | 1.03 (0.99–1.07) | 0.162 | 1.17 (0.77–1.78) | 0.466 |  | 1.03 (1.00–1.07) | 0.074 |
| Neonatal death |  | 1.01 (0.96–1.06) | 0.716 | 1.41 (0.70–2.84) | 0.336 |  | 1.04 (1.00–1.08) | 0.048 |
| Early neonatal death |  | 1.06 (1.00–1.12) | 0.048 | 2.72 (0.97–7.60) | 0.056 |  | 1.08 (1.03–1.14) | 0.001 |
| Late neonatal death |  | 0.97 (0.91–1.03) | 0.273 | 0.49 (0.18–1.33) | 0.161 |  | 0.99 (0.94–1.04) | 0.741 |
| Post-neonatal death |  | 1.06 (0.99–1.13) | 0.112 | 1.02 (0.58–1.77) | 0.952 |  | 1.00 (0.95–1.06) | 0.858 |
| ^a^ adjusted for birth order, birthweight pair difference, birthweight pair mean, gestational age and male sex by gestational age (interactive term). | | | | | | | | |

| **Table S8:** Association between male sex and mortality outcomes, including interaction between gestational age and birthweight pair mean | | | | | |
| --- | --- | --- | --- | --- | --- |
|  |  | **aOR** ^a^  **(95% CI)** | ***P*** |  | **Interactive term** ^b^ ***P*** |
| **Outcomes** |  |  |  |  |  |
|  |  |  |  |  |  |
| Infant death |  | 1.46 (1.26–1.71) | <0.001 |  | <0.001 |
| Neonatal death |  | 1.40 (1.18–1.67) | <0.001 |  | <0.001 |
| Early neonatal death |  | 1.51 (1.23–1.85) | <0.001 |  | <0.001 |
| Late neonatal death |  | 1.09 (0.85–1.40) | 0.511 |  | <0.001 |
| Post-neonatal death |  | 1.45 (1.12–1.88) | 0.004 |  | 0.285 |
| ^a^ Adjusted for birth order, birthweight pair difference, birthweight pair mean, gestational age and interactive term; ^b^ Birthweight pair mean by gestational age. | | | | | |

| **Table S9:** Interaction between male sex and birth order in the association between male sex and mortality outcomes, separately for first and second born twins | | | | | | | | |
| --- | --- | --- | --- | --- | --- | --- | --- | --- |
| **Outcomes** |  | **First born** | | | **Second born** | |  | **Interactive term** ^b^ |
|  |  | **aOR** ^a^  **(95% CI)** | ***P*** |  | **aOR** ^a^  **(95% CI)** | ***P*** |  | ***P*** |
|  |  |  |  |  |  |  |  |  |
| Low Apgar1 |  | 1.00 (0.89–1.12) | 0.994 |  | 1.07 (0.96–1.18) | 0.23 |  | 0.476 |
| Low Apgar5 |  | 1.10 (0.84–1.44) | 0.501 |  | 1.33 (1.05–1.70) | 0.02 |  | 0.35 |
| Congenital anomalies |  | 1.80 (1.34–2.41) | <0.001 |  | 1.42 (1.07–1.89) | 0.015 |  | 0.288 |
| Infant death |  | 1.15 (0.92–1.44) | 0.207 |  | 1.65 (1.35–2.03) | <0.001 |  | 0.025 |
| Neonatal death |  | 1.11 (0.85–1.45) | 0.429 |  | 1.59 (1.25–2.02) | <0.001 |  | 0.069 |
| Early neonatal death |  | 1.28 (0.94–1.74) | 0.111 |  | 1.57 (1.19–2.09) | 0.002 |  | 0.366 |
| Late neonatal death |  | 0.78 (0.52–1.18) | 0.235 |  | 1.38 (0.98–1.96) | 0.066 |  | 0.04 |
| Post-neonatal death |  | 1.22 (0.85–1.75) | 0.275 |  | 1.77 (1.22–2.56) | 0.003 |  | 0.162 |
| ^a^ adjusted for birth order, birthweight pair difference, birthweight pair mean, gestational age and interactive term; ^b^ male sex by birth order | | | | | | | | |

| **Table S10:** Interactions of male sex with standardised birthweight by gestational age for twins (A) and male sex with appropriate birthweight for twins (standardised birthweight >= 0) (B) in the association between male sex and mortality outcomes | | | | | | |
| --- | --- | --- | --- | --- | --- | --- |
| **Outcomes** |  | **A** ^a^ | | | **B** ^c^ | |
|  |  | **Interactive term** ^b^  **(95% CI)** | ***P*** |  | **Interactive term** ^d^  **(95% CI)** | ***P*** |
|  |  |  |  |  |  |  |
| Infant death |  | 0.99 (0.87–1.12) | 0.849 |  | 0.90 (0.67–1.21) | 0.469 |
| Neonatal death |  | 1.03 (0.90–1.18) | 0.725 |  | 0.86 (0.61–1.22) | 0.398 |
| Early neonatal death |  | 1.06 (0.90–1.25) | 0.477 |  | 1.11 (0.75–1.64) | 0.612 |
| Late neonatal death |  | 0.98 (0.78–1.22) | 0.838 |  | 0.58 (0.35–0.98) | 0.042 |
| Post neonatal death |  | 0.89 (0.71–1.12) | 0.309 |  | 0.96 (0.58–1.61) | 0.890 |
| a Adjusted for birth order, standardised birthweight by gestational age, gestational age and interactive term; ^b^ male sex by standardised birthweight per gestational age; ^c^ Adjusted for birth order, appropriate birthweight (standardised birthweight > 0), gestational age and interactive term; ^d^ male sex by appropriate birthweight. Note: Standardisation of the birthweight variable was done using twin-specific birthweight percentiles found in Min et al. (2000), see Methods section for details. | | | | | | |
